# Cognitive function in bipolar disorder

**DOI:** 10.1192/j.eurpsy.2022.426

**Published:** 2022-09-01

**Authors:** T. Sparding

**Affiliations:** The Institute of Neuroscience and Physiology, Department Of Phychiatry Andneurochemistry, Göteborg, Sweden

**Keywords:** cognition, bipolar disorder, Longitudinal study, functioning

## Abstract

**Introduction:**

In bipolar disorder, cognitive deficits persist across mood episodes and euthymia. Despite recent advances, cognitive impairment in bipolar disorder remains poorly understood. The presentation will focus on recent work where different approaches are used to clarify the role of cognitive deficits in bipolar disorder.

**Objectives:**

First, we have examined the clinical relevance of cognitive impairments and examined if cognitive abilities differ between bipolar disorder subtypes and healthy controls. Second, we examined if cognitive abilities differ between individuals with bipolar disorder with and without attention-deficit hyperactivity disorder. Third, we examined the relationship between cognitive functioning and occupational functioning. Lastly, we examined if long-term changes in cognitive functioning in bipolar disorder patients differ from normal aging.

**Methods:**

The St. Göran Bipolar Project is an interdisciplinary, prospective, naturalistic study of bipolar disorder. Patients were recruited and followed-up at two specialized out-patient clinics in Stockholm and Gothenburg, Sweden.

**Results:**

We showed that there is evidence for significant cognitive heterogeneity in bipolar disorder. Comorbid ADHD could not explain this heterogeneity. Moreover, we showed that excutive functioning is a powerful predictor of occupational functioning. The cognitive trajectory over a 6-year period did not differ between bipolar disorder patients and healthy controls.

**Conclusions:**

There is no conclusive cognitive profile characterizing bipolar disorder. However, cognitive functioning is of great importance in understanding occupational functioning in bipolar disorder. Contrary to the assumption that cognitive impairments may be progressive we show that changes in cognitive functioning over time do not differ between patients and healthy controls.

**Disclosure:**

No significant relationships.

